# Effects of 12-month home-based physiotherapy on duration of living at home and functional capacity among older persons with signs of frailty or with a recent hip fracture - protocol of a randomized controlled trial (HIPFRA study)

**DOI:** 10.1186/s12877-018-0916-y

**Published:** 2018-10-01

**Authors:** Paula Soukkio, Sara Suikkanen, Sanna Kääriä, Hannu Kautiainen, Sarianna Sipilä, Katriina Kukkonen-Harjula, Markku Hupli

**Affiliations:** 1 Rehabilitation, South Karelia Social and Health Care District, Valto Käkelän katu 3, FI-53130 Lappeenranta, Finland; 2 Gerontology Research Center, Unit of Health Sciences, University of Jyväskylä, Rautpohjankatu 8, FI-40700 Jyväskylä, Finland; 3 Department of General Practice and Helsinki University Hospital, University of Helsinki, Unit of Primary Health Care, Tukholmankatu 8 B, FI-00290 Helsinki, Finland; 4 Aureolis Oy, Hevosenkenkä 3, FI-02600 Espoo, Finland

**Keywords:** Frail elderly, Hip fractures, Community-dwelling, Home-based physiotherapy, Duration of living at home, Functional capacity, Social and health care services

## Abstract

**Background:**

Health concerns, such as frailty and osteoporotic fractures decrease functional capacity and increase use of health and social care services in the aging population. The ability to continue living at home is dependent on functional capacity, which can be enhanced by rehabilitation.

We study the effects of a 12-month home-based physiotherapy program with 12-month follow-up on duration of living at home, functional capacity, and the use of social and health care services among older persons with signs of frailty, or with a recently operated hip fracture.

**Methods:**

This is a non-blinded, parallel group, randomized controlled trial performed in South Karelia Social and Health Care District, Finland (population 131,000). Three hundred community-dwelling older persons with signs of frailty (age ≥ 65) and 300 persons with a recent hip fracture (age ≥ 60) will be recruited. Frailty is screened by FRAIL questionnaire and verified by modified Fried’s frailty criteria. Both patient groups will be randomized separately to a physiotherapy and a usual care arm. Individualized, structured and progressive physiotherapy will be carried out for 60 min, twice a week for 12 months at the participant’s home. The primary outcome at 24 months is duration of living at home. Our hypothesis is that persons assigned to the physiotherapy arm will live at home for six months longer than those in the usual care arm. Secondary outcomes are functional capacity, frailty status, health-related quality-of-life, falls, use and costs of social and health care services, and mortality. Assessments, among others Short Physical Performance Battery, Functional Independence Measure, Mini Nutritional Assessment, and Mini-Mental State Examination will be performed at the participant’s home at baseline, 3, 6, and 12 months. Register data on the use and costs of social and health care services, and mortality will be monitored for 24 months.

**Discussion:**

Our trial will provide new knowledge on the potential of intensive, long-term home-based physiotherapy among older persons at risk for disabilities, to enhance functional capacity and thereby to postpone the need for institutional care, and diminish the use of social and health care services.

**Trial registration:**

ClinicalTrials.gov Identifier: NCT02305433, Registered Nov 28, 2014.

## Background

In the aging population, one of the major burdens in social and health care is frailty. Frailty is a multidimensional condition, related to poor resolution of homeostasis after stressor events [1–3]. It can lead to increased vulnerability, disability, risk of falling, need for long-term care, mortality [1–3], and is associated with increased health care costs [4]. Hip fractures are another major burden causing morbidity, impairments, increased need for care, and mortality [5–7]. The yearly economic burden of hip fractures in Europe alone is estimated to be over 16 billion euros [7].

In persons over 65 years the prevalence of frailty varies depending on the criteria used, being from 4 to 59% [8, 9]. The most common definition is based on the frailty phenotype [1], which includes five criteria: unintentional weight loss, exhaustion, physical inactivity, slow walking speed, and weak grip strength. The prevalence of frailty increases with age and is higher among women than among men [10]. Both frailty and pre-frailty states are significant predictors of nursing home placement [11].

Worldwide the incidence of hip fractures varies widely and is highest in North Western Europe [12]. In western countries 10–20% of the persons are institutionalized when evaluated 6–12 months after the hip fracture [13], and 11% change their dwelling (from home to institution or vice versa) during 4–12 months after the fracture [14]. At one year since the fracture 42% of the patients have not recovered to their pre-fracture mobility level, and 29% do not achieve the same level of activities of daily living that they had before the fracture [15]. Excess mortality during the first post-fracture year of hip fracture patients is 8–36% [16]. Frailty is common among hip fracture patients [17], and the frailty status influences the recovery [18].

Physical activity has beneficial effects on muscle strength and endurance in older adults [19]. Frailty is an indication to start physical exercise [19] to improve functional capacity [20]. Multicomponent exercise interventions (including aerobic, strength, balance and flexibility exercises) reduce the incidence, prevalence and severity of frailty [21, 22], and enhance functional capacity [18, 23] and overall mobility [24] of hip fracture patients.

To our knowledge, long-term home-based physiotherapy and whether it can prolong duration of living at home have not been studied extensively in community-dwelling older persons with signs of frailty or with a recently operated hip fracture. Our objective is to study the effects of a structured 12-month home-based physiotherapy program with 12-month follow-up on duration of living at home, functional capacity, and the use of social and health care services. The main hypothesis is that home-based physiotherapy lengthens the duration of living at home by six months as compared to usual care in both patient groups. We also hypothesize that physiotherapy improves functional capacity, and decreases the use and costs of social and health care services.

## Methods

### Design

**Fig. 1:**
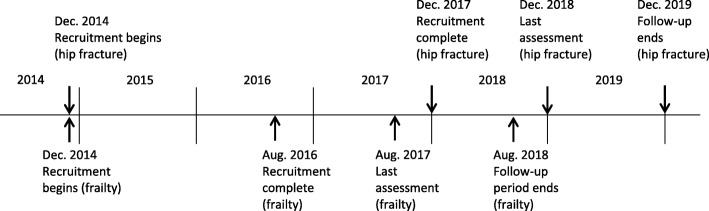
Timetable

HIPFRA study is a parallel group, non-blinded, randomized controlled trial carried out in Finland, in South Karelia Social and Health Care District (Eksote), population 131,000, of whom 25% were 65 years or older in 2015. The study lasts for 24 months and has two phases; 1) home-based physiotherapy intervention for the first 12 months, and 2) registry follow-up for the second 12 months. The study has started in December 2014 and the final follow-up period ends in December 2019. For a more detailed timetable, see Fig. 1.

### Outcomes of the study

The primary outcome is duration of living at home. The secondary outcomes are functional capacity, frailty status, health-related quality-of-life, and number of falls, use and costs of social and health care services, and mortality.

### Participants and eligibility criteria

The target population is older adults with signs of frailty or with a recently operated hip fracture, who live at home, are able to walk indoors without or with mobility aids, and are able to communicate in Finnish.

For persons with signs of frailty the inclusion criteria are: 1) one or more points from FRAIL questionnaire [25] (screening), 2) one or more points from modified Fried et al.’s frailty criteria [1] (verification), 3) age 65 or older, and 4) Mini-Mental State Examination (MMSE) [26] score ≥ 17. For hip fracture patients the inclusion criteria are 1) ICD-10 diagnosis codes S72.0, S72.1, S72.2, 2) the index fracture is the first operated one in the present hip, 3) age 60 or older, and 4) Mini-Mental State Examination (MMSE) [26] score ≥ 12.

Exclusion criteria for both patient groups are: 1) resident in institutional care, 2) advanced diseases which prevent from participating in long-term physiotherapy, such as severe neurological diseases (e.g. multiple sclerosis, cerebrovascular disorders), cardiovascular diseases with severely impaired physical capacity (NYHA class III or IV), severe musculoskeletal diseases (e.g. severe rheumatoid arthritis), or severe or acute mental problems (major depression, psychosis or schizophrenia), 3) alcohol or drug abuse, 4) severe problems with hearing or eyesight, or 5) terminal illnesses.

### Randomization

A computer-generated random allocation sequence was prepared by a statistician at University of Helsinki. The statistician is not involved in the management of patients. The two patient groups are randomized separately to a home-based physiotherapy and a usual care arm. Random allocation is performed by the project manager using the computer program, in consecutive order after baseline assessments. The randomization block size varies from 2 to 10 and the sequence is concealed. The project manager informs the participant by telephone about the allocation result. If the participant is allocated to the physiotherapy arm, the manager also informs the physiotherapist by telephone about the new rehabilitee, and physiotherapy starts within a week.

### Recruitment

#### Recruitment of persons with signs of frailty

**Fig. 2:**
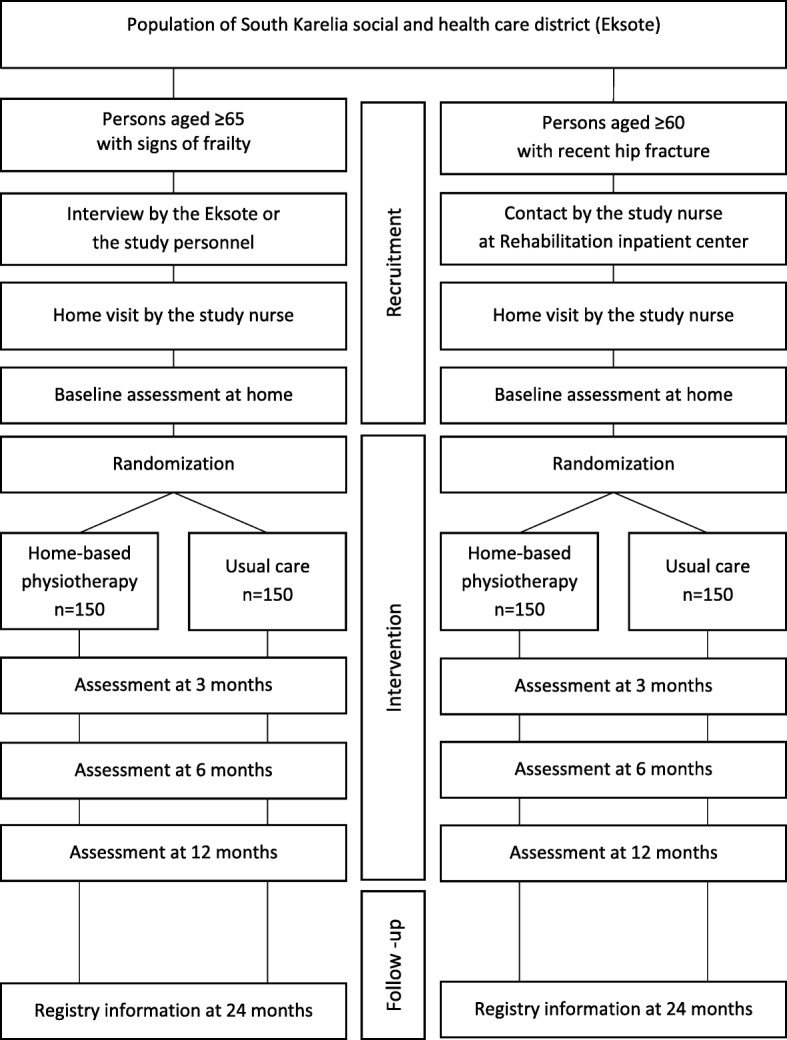
Flow chart

Potential participants are informed about the study by advertisements in local newspapers or by Eksote personnel. The recruitment process has two phases (Fig. 2). First, frailty screening is carried out using FRAIL questionnaire [25] either in face-to-face contacts or over the phone by Eksote personnel. In the second phase, the study nurse visits the person’s home, gives oral and written information about the study, checks that all the inclusion criteria and none of the exclusion criteria are fulfilled, and verifies the frailty status using modified Fried’s criteria [1]. The criteria were modified by assessing physical activity using the question on frequency of physical activity according to Health Behaviour and Health of the Finnish Elderly survey [27], and by using a cut-off value of 8.7 s for the 4-m walking time, which is based on the lowest quarter for persons aged 71 and older in Short Physical Performance Battery [28].

#### Recruitment of persons with a recent hip fracture

Recruitment begins at Eksote Rehabilitation Inpatient Center, to which the majority of the hip fracture patients are transferred from Eksote orthopedic wards in less than a week after surgery. The study nurse meets possibly eligible patients in the rehabilitation inpatient center and gives them oral and written information about the study. For those who are interested in participating, a home visit is performed by the nurse within a week after discharge. The content of the home visit is the same as for the persons with signs of frailty.

### Assessments

Previously validated methods for data collection are used in HIPFRA study. Information about the main outcome, duration of living at home, is retrieved from the electronic health records (EHR) of Eksote. The duration of living at home is defined as number of days lived at home during 24 months. Days spent in hospital or in institutional care reduces the number of days lived at home. Change to reside permanently in institutional care or death (within 24 months) are defined as end points.

Interviews, questionnaires, measurements and register information are used to collect data for secondary outcomes. Measurements and interviews based on structured questionnaires are performed at baseline, and after 3, 6, and 12 months at the participant’s home by the study physiotherapist or the study nurse, who are not blinded to randomization. The visit lasts approximately for one and half hours. Before the baseline assessment the participant signs a written informed consent. Before visits the participants are mailed self-reported questionnaires. Register information (for 0–24 months) on the use and costs of social and health care services, medication and mortality will be retrieved from Eksote EHR, the Population Register Centre, and the registers of the Social Insurance Institution (for 0–24 months). Assessment methods of outcomes and other parameters, and the assessment schedule during 24 months are presented in Table 1.

**Table 1:** Outcomes, assessment methods and schedule

Outcomes	Parameters and measures [reference]	Scale	Assessment method	Assessment time points (months from the beginning)				
				0	3	6	12	24
Primary outcome	Duration of living at home (days) during 24 months	1–730	Register information					x
Secondary outcomes								
Functional capacity	Instrumental Activities of Daily Living (IADL) [54]	8-31	Interview based on a structured questionnaire	x	x	x	x	
	Functional Independence Measure (FIM) [55]	18–126	Interview based on a structured questionnaire	x	x	x	x	
	Short Physical Performance Battery (SPPB) [28, 56]	0–12	Measurement	x	x	x	x	
	Grip strength (Saehan dynamometer, model Sh5001, South Korea) [57]		Measurement	x	x	x	x	
Frailty status	Fried et al.’s frailty criteria (modified) [1]	0–5	Interview based on a structured questionnaire and measurement	x			x	
Quality-of-life	Health-related quality-of-life (HRQoL) (15D©) [58, 59]	15–75	Self-reported questionnaire	x	x	x	x	
Falls	Number of falls		Interview based on a structured questionnaire	x	x	x	x	
Service use and costs	Use and costs of social and health care services, and medication		Register data (0–12 months and 13–24 months) from EHR and Social Insurance Institution				x	x
Mortality	Mortality		Register data from EHR					x
Other outcomes								
Cognition	Mini-Mental State Examination (MMSE) [26]	0–30	Interview based on a structured questionnaire	x		x	x	
Depressive symptoms	Geriatric Depression Scale (GDS-15) [60]	0–15	Interview based on a structured questionnaire	x	x	x	x	
Nutritional status	Mini Nutritional Assessment (MNA) [38]	0–30	Interview based on a structured questionnaire	x	x	x	x	
Fear of falling	Falls Efficacy Scale International (FES-I) [61, 62]	16–64	Interview based on a structured questionnaire	x	x	x	x	
Social support	Social Provision Scale (SPS) [63]	24–96	Self-reported questionnaire	x		x	x	
Health status	Weight (Omron HN289, Japan)		Measurement	x	x	x	x	
	Height (KaWe PERSON-CHECK, Germany)		Measurement	x	x	x	x	
	BMI		Calculated from weight and height	x	x	x	x	
	Diseases diagnosed by a physician		Interview based on a structured questionnaire and EHR	x			x	
	Medication		Interview based on a structured questionnaire and EHR	x	x	x	x	
	Pain (Visual Analogue Scale, VAS) [64]	0–100	Interview based on a structured questionnaire	x	x	x	x	
	Medical and mobility aids		Interview based on a structured questionnaire and EHR	x	x	x	x	
	Perceived health [27]		Interview based on a structured questionnaire	x	x	x	x	
	Perceived mobility and physical fitness [27]		Interview based on a structured questionnaire	x	x	x	x	
	Hip fracture surgery (details of operation, inpatient rehabilitation)		EHR	x				
Home services	Home care and home health care		Interview based on a structured questionnaire and EHR	x	x	x	x	
	Home renovations		Interview based on a structured questionnaire and EHR	x	x	x	x	
Lifestyle	Physical activity [27]		Interview based on a structured questionnaire	x	x	x	x	
	Alcohol consumption (AUDIT-C) [65]	0–12	Interview based on a structured questionnaire	x	x	x	x	
	Smoking [27]		Interview based on a structured questionnaire	x	x	x	x	
Background information	Demographic factors (age, marital, status, education, previous occupation)		Interview based on a structured questionnaire	x				
	Type of dwelling and household		Interview based on a structured questionnaire	x	x	x	x	

### Home-based physiotherapy intervention

The goal is to restore and increase the participant’s functional capacity, especially in activities of daily living, to prolong duration of living at home. One supervised physiotherapy session at the participant’s home lasts for 60 min, and takes place twice a week for 12 months. The participant has the same physiotherapist throughout the year. Physiotherapy is structured and progressive and consists of multicomponent exercises (Table 2). An individual physiotherapy plan is prepared by the physiotherapist in cooperation with the participant. The goal setting is done by using the Goal Attainment Scaling (GAS) method [29, 30]. Goals are evaluated and reported after 3, 6 and 12 months of therapy by the physiotherapist and the participant. The dates, duration, content (type, intensity, perceived exertion, and the number of sets and repetitions), and any adverse effects of each physiotherapy session are reported monthly by the physiotherapist.

**Table 2:** Contents of home-based physiotherapy

	Warm-up	Strength exercises	Flexibility exercises	Balance exercises	Functional exercises
Modes of activities	Various activities	Orientation 2–3 weeks: based on the Otago exercise program [31] (5 leg muscle strengthening exercises with up to 4 levels of difficulty). Muscle strength, power and endurance periods, each 8 weeks, repeated twice during 12 months	Various flexibility exercises for large joints and the spine to enlarge the ROM	Otago [31] exercises (12 balance exercises with up to 4 levels of difficulty)	Flexibility, strength and balance exercises combined with IADL activities and walking outside
Intensity	Low to moderate	Moderate to vigorous (12–17 of RPE) [36]	Moderate	Challenges to the individual’s balance abilities	Challenges to the individual’s functional abilities
Progression	Changing intensity and activities according to physical condition	Using extra weights, increasing the level of difficulty	Changing activities	Selecting more advanced balance exercises (e.g. static, dynamic, dual task)	Selecting more advanced functional exercises
Frequency	2 times / week	2 times / week	2 times / week	2 times / week	One time / week
Duration	Approx. 5–10 min	Approx. 30 min	Approx. 10 min	Approx. 20 min	Integrated to other exercises
Assessment	Shortness of breath	SPPB [28, 56], RPE [36]	ROM	Time (s), observation	Task accomplishment

*IADL* Instrumental Activities of Daily Living, *ROM* Range of motion, *RPE* Rating of Perceived Exertion (scale 6 to 20), *s* second(s), *SPPB* Short Physical Performance Battery

#### Exercises for muscle strength, power and endurance

The goal is to enhance the participant’s muscle strength, power and endurance, especially in lower limbs, in order to improve postural balance, ability to walk, and functional capacity. Physiotherapy starts with a 2–3-week orientation period based on the Otago exercise program [31]. Strength training is progressive, and follows a cycle where 8-week periods of strength, power and endurance exercises alternate during the intervention year. The intensity goal of strength exercises is 60–80% from the person’s maximal strength of the trained muscle [32–34]. Sets per muscle vary from 2 to 5, and repetitions per set vary from 3 to 12. Muscle power exercises are performed with low to moderate intensity (20–60%) [32], and with high velocity of movement. In power exercises approximately 3 sets and repetitions from 4 to 10 are used [35]. Endurance exercises consist of at least 12 and up to 15–30 repetitions per set, and 2–3 sets per muscle, and the intensity is 20–60% from the person’s maximal strength of the trained muscle [34].

Strength training is individually tailored, and training sets and repetitions are modified according to the participant’s performance [32], health status, and perceived exertion rated by the Borg Rating of Perceived Exertion Scale (RPE, scale 6 to 20) [36] during every session. Progression is achieved by adding resistance with extra weights, such as ankle weights, weight vests, kettlebells and dumbbells, and modifying the sets and repetitions during 12 months.

#### Balance and flexibility exercises

The goal for balance exercises is to enhance the participant’s functional capacity and ability to walk in order to prevent falls. Balance training begins with the Otago exercise program [31], and later on functional exercises are used. The goal for flexibility exercises is to enhance the participant’s range of motion especially in ankle joints and in large joints to maintain activities of daily living. Dynamic flexibility exercises are performed in every physiotherapy session as warm-up exercises or combined with functional exercises.

#### Functional exercises

Functional exercises, such as climbing stairs, chair rise, and walking outside are used to train muscle strength and endurance. Activities of daily living, such as washing dishes, preparing meals, doing laundry or cleaning up are used to train flexibility and balance abilities. Functional exercises are more effective than resistance exercises to improve functional task performance [37].

#### Counselling on physical activity and nutrition

The physiotherapist encourages the participant to exercise on their own, and if possible to take part in the exercise groups organized by municipalities or by third sector organizations. Brief nutritional counselling by the physiotherapist is based on the MNA test [38] and national nutritional guidelines. The main goal of guidance is to reverse possible malnutrition, and ensure sufficient protein intake to prevent weight loss and sarcopenia. Oral nutritional supplements are recommended if necessary.

### Usual care

The participants in the usual care arm continue to live their life “as usual”. They can get any social or health care services that they may need during the 24 months they participate in the study.

### Statistical analyses

The statistical power calculations are based on the hypothesis that the persons with hip fracture assigned to the physiotherapy arm will live at home for six months (180 days) longer than those in the control arm when assessed at 24 months. Our calculations are based on the Finnish PERFECT (PERFormance, Effectiveness and Cost of Treatment episodes) data on hip fractures [39], in which data are available on the proportion of patients living at home 1 year after the fracture (in 2005). To detect a difference of 180 days between the arms, when the type I error is set at 0.05 and the statistical power at 0.8, a sample size of 91 persons in each arm is needed. To allow for discontinuation (estimated as 15% of the participants) and death of patients (20%) during our study period of 2 years, our targeted sample is 300 patients. As to the power calculations of patients with signs of frailty, there are no previous data on the duration of living at home. Therefore we use the same power calculations as for patients with hip fracture. However, the mortality of frail patients is assumed to be lower than that of hip fracture patients.

Frequencies, means and standard deviations will be used to describe background variables of the participants. Differences between the physiotherapy and usual care arm at baseline will be tested by chi-squared test or Fisher exact test for categorical variables, and by t-test or bootstrap type test for continuous variables as appropriate. Analyses will be carried out according to intention to treat. Repeated data will be analyzed using generalized estimating equations (GEE) models with appropriate distribution and link function. In the case of violation of the assumptions (e.g. non-normality), a bootstrap-type test will be used. The normality of the variables will be tested by using the Shapiro-Wilk W test. Incidence rates with 95% confidence intervals (CI) will be calculated assuming a Poisson distribution. Crude and standard estimates of incidence rate ratios (IRR) will be calculated using Poisson regression models, or negative binomial regression models when appropriate. Generalized linear regression model with log link and gamma variance functions will be estimated for social and health service use and costs. Variance function will be selected based on Park test and Akaike’s information criterion. Mortality and its risk factors will be assessed with Cox proportional hazard regression models. Analyses will be performed using IBM SPSS statistics 24 software.

### Ethical issues

During recruitment, comprehensive oral and written information of the study is given to persons interested in participating in the study. Participation is voluntary. If they are eligible and willing, they sign an informed consent before the baseline assessment. All data collected will be recorded, stored and reported anonymously. This study was approved by an independent coordinating ethics committee in Helsinki University Hospital (HUS). The study was prospectively registered in ClinicalTrials.gov (NCT02305433) on November 28, 2014.

## Discussion

We study the effects of long-term home-based physiotherapy in community-dwelling older persons with signs of frailty and recently operated hip fracture in randomized settings to postpone institutionalization. Our main interests are on the duration of living at home, functional capacity, and the use and costs of social and health care services.

In older persons with signs of frailty, interventions using physical exercises have improved functional outcomes such as gait speed and SPPB score [40]. However, the results on quality-of-life [41] or on balance and ADL functions [40] are not consistent. In community-dwelling older adults, exercise interventions have been effective in reducing or preventing frailty [42]. Persons with signs of frailty seem to benefit from multicomponent exercise programs, but the optimal program content still remains unclear [43, 44]. In persons with signs of frailty, individually instructed and supervised exercise has been more effective on physical functioning than group exercises [41]. The duration of therapy has most often lasted for three months (from 1 to 18 months), and interventions for at least 5 months had better outcomes than shorter ones [45].

After hip fracture, ordinary postoperative care is not usually sufficient to maximize performance recovery [18], as recuperation time for balance and gait can be up to 9 months and for walking speed up to 11 months [46]. Extended exercise programs have had positive impact on functional capacity [47], and reducing or reversing disability of hip fracture patients [18, 23]. Specifically, extended exercise programs outside the hospital (e.g. at home) improve physical functioning [24, 47]. Similarity to persons with signs of frailty, there is also evidence for hip fracture patients that individualized, multicomponent and progressive rehabilitation enhance functional capacity [18, 23].

The physiotherapy intervention in our randomized study lasts for 12 months with maximally 104 physiotherapy sessions. Participation for 12 months in physiotherapy requires strong commitment, motivation and resiliency from the patients. In Baltimore hip fracture studies [48, 49] on average 44 of 56 home exercise sessions were completed during 12 months. This implies that it is possible to engage frail older persons with hip fracture to a year-long home-based exercise intervention [49]. Physiotherapy performed at home can enhance training adherence for older persons with lower functional capacity [48], because no travelling is required. Interventions with longer durations have usually more dropouts than shorter ones [45]. On the other hand, supervised training and long-term physiotherapy relationship between the physiotherapist and the participant may improve the participant’s motivation and attitude towards training, which enables progression and increased training intensity [50].

Cognitively impaired patients are often excluded from rehabilitation studies [51] even if there is evidence that for example, hip fracture patients with mild or moderate dementia [52] or community-dwelling persons with Alzheimer’s disease [53] clearly benefit from intensive geriatric rehabilitation. This is why we accept mildly cognitively impaired persons (MMSE ≥17 points) in our frailty group, and moderately cognitively impaired persons (MMSE ≥12) in our hip fracture group.

During the last decade, the focus in social and health care services for older persons in Eksote and generally in Finland has changed from facility-based services to services at home to enhance longer community-dwelling. Thus there is a need to study the effects and cost-effectiveness of home-based services, e.g. physiotherapy. In this pragmatic trial we implement long-term home-based physiotherapy intervention and evaluate it with repeated comprehensive assessments at home. We apply screening tools to identify community-dwelling persons with signs of frailty, who might benefit from long-term physiotherapy. Later on, frailty screening could be used in primary health care to identify community-dwelling persons in need for more intense services in order to prevent deterioration, loss of functional capacity and institutionalization.

HIPFRA study will provide new knowledge on whether an individualized, multicomponent, long-term and supervised home-based physiotherapy can improve functional capacity, diminish the degree of frailty, prolong living at home by postponing institutional care, and diminish the use of social and health care services and decrease their costs in persons with signs of frailty and in persons with recently operated hip fracture.

## Abbreviations

15D©: 15-dimensional health-related quality-of-life instrument
AUDIT-C: Alcohol Use Disorders Identification Test
EHR: Electronic health records
Eksote: South Karelia Social and Health Care District
FES-I: Falls Efficacy Scale International
FIM: Functional Independence Measure
FRAIL: Fatigue, Resistance, Ambulation, Illnesses, & Loss of Weight
GDS-15: Geriatric Depression Scale
HIPFRA: Home physiotherapy for HIP fracture and FRAilty
HRQoL: Health-related quality-of-life
IADL: Instrumental Activities of Daily living
MMSE: Mini-Mental State Examination
MNA: Mini Nutritional Assessment
NYHA: New York Heart Association
SPPB: Short Physical Performance Battery
SPS: Social Provision Scale
VAS: Visual Analogue Scale
